# Brief and long maternal separation in C57Bl6J mice: behavioral consequences for the dam and the offspring

**DOI:** 10.3389/fnbeh.2023.1269866

**Published:** 2023-10-23

**Authors:** Cynthia Rombaut, David Roura-Martinez, Catherine Lepolard, Eduardo Gascon

**Affiliations:** Aix Marseille Univ, CNRS, INT, Inst Neurosci Timone, Marseille, France

**Keywords:** maternal separation, maternal behavior, mice, behavior, early life stress (ELS)

## Abstract

**Introduction:**

Animal models, especially rodents, have become instrumental to experimentally investigate the effects of an adverse post-natal environment on the developing brain. For this purpose, maternal separation (MS) paradigms have been widely used in the last decades. Nonetheless, how MS affects maternal behavior and, ultimately, the offspring depend on multiple variables.

**Methods:**

To gain further insights into the consequences of MS, we decided to thoroughly measure and compare the effects of short (15 min, 3 times/day) vs. long (3 h, 1 time/day) separation on multiple maternally-associated behaviors and across the entire post-natal period.

**Results:**

Compared to unhandled control litters, our results confirmed previous studies and indicated that SMS enhanced the time and variety of maternal care whereas LMS resulted in poor caregiving. We also showed that SMS-accrued caregiving persisted during the whole post-natal period. In contrast, LMS effects on maternal behavior were restricted to the early life (P2-P10). Finally, we also analyzed the behavioral consequences of these different rearing social environments on the offspring. We found that MS has profound effects in social tasks. We showed that affiliative touch, a type of prosocial behavior that provides comfort to others, is particularly sensitive to the modification of maternal caregiving.

**Discussion:**

Our results provide further support to the contention that interactions during the early post-natal period critically contribute to emotional processing and brain co-construction.

## Introduction

It has been long acknowledged that environment during early life fine-tunes brain development and has long-lasting consequences on its function. Thus, classical studies on sensory deprivation (Wiesel and Hubel, 1963; Belford and Killackey, 1980) led to the identification of critical periods, defined as time windows in which neural circuits are critically shaped by experience. Those periods allow individuals to adapt their behaviors to their actual environment.

In humans, social environment, especially parental care, is a potent effector of brain development. It is now firmly established that impairments in early caregiving, such as exposure to violence, neglect or abuse consistently associate with altered neurodevelopment and psychopathology (Felitti et al., 1998; Edwards et al., 2003; Carr et al., 2013). A large animal literature has confirmed the deleterious effects on brain function of disrupting the normal interactions between offspring and mother (Levine, 1967; Romeo et al., 2003; Callaghan and Richardson, 2011). More importantly, mice having experienced adverse caregiving show an enhanced vulnerability to adult chronic stress and develop depressive-like behaviors (Peña et al., 2017, 2019a; Alizadeh-Ezdini and Vatanparast, 2022).

Although multiple paradigms have been set up for studying the impact of early adverse life experience in rodents, maternal separation (MS) has been widely applied. Nonetheless, MS findings are still controversial mainly because of methodological discrepancies and underpowered designs (Tractenberg et al., 2016; Bonapersona et al., 2019; Schuler et al., 2022). Inconsistencies in MS protocols may result in profound differences in the effects on both offspring and dams (Lehmann and Feldon, 2000; Tractenberg et al., 2016). Thus, the effects of MS on dam behavior depend on the separation duration (Orso et al., 2019); some groups (but not all) have reported that short bouts of separation (around 15 min/day) result in a robust augmentation of maternal behaviors on reunification of pups and dams (i.e., pup retrieval or licking) (Korosi et al., 2010; Singh-Taylor et al., 2018). In contrast, longer separation times (>1 h/day) induce, in some studies, a significant disruption of the dam-pups relationship (Plotsky and Meaney, 1993; Peña et al., 2019a; Kestering-Ferreira et al., 2021). Similarly, it has been suggested that effects of MS are time-dependent as only separation during late post-natal phases (P10–P20 in mice) affected the vulnerability to adult chronic stress (Peña et al., 2019a,b). Finally, some studies have combined MS and other stressors (such as limiting bedding or nesting material) further complicating the interpretation of existing results (Peña et al., 2017; Orso et al., 2020). Given the relevance of pup-dam interactions for the development of social and emotional functions, it is critical to thoroughly describe how different MS protocols impinge on maternal care levels.

Despite a growing body of evidence linking MS and anxiety/depression-like behaviors, very few studies have investigated the effects of a positive rearing environments. For these reasons, we believe that our study will help the field of MS to move forward by expanding our understanding of maternal behavior under different MS conditions as well as their long-term consequences on the offspring. To do this, we sought to: (i) quantitatively characterize maternal behaviors under three experimental conditions: short MS (15 min, 3 times/day) mimicking the effects of dam absences for food seeking, long MS (3 h, 1 time/day) to model ‘traumatic’ separation and undisturbed controls; (ii) provide a detailed time course of maternal care over the complete post-natal period (from birth to weaning); and (iii) investigate how exposure to distinct maternal caregiving environments can lead to far-reaching alterations in behavioral outcomes independently of adult stress.

## Methods

### Animals

To facilitate replication of our findings, our mice were not bred in-house but purchased to Charles Rivers (France). Animals were kept in the local animal facility, under controlled temperature and a 12 h/12 h light–dark cycle (lights on at 7 AM to 7 PM). Animals had access to food and water *ad libitum* during the whole experiment.

Pregnant C57Bl6J females arrived at the local animal facility at E15 in order to facilitate accommodation to the novel environment and release any stress associated with transportation before delivery. 3–4 days later, females give birth (postnatal day (PND) 0) and pups from different litters were mixed and randomly assigned to an experimental group (CT). To limit variability in litter size, each female to be studied was isolated in a recording unit (Phenotyper, Noldus) with 6 pups. Litters were weighted every 2 days during MS protocol. To provide a homogeneous social context after weaning (PND21), each mouse was caged with an old non-reproductive CD1 female. Cleaning was performed once per week. All experiments were performed during the light phase (9 am to 7 pm) under standard illumination (around 300 lx).

All procedures involving mice were approved by the local ethics committee and are in agreement with European regulations (Directive 2010/63/EU). A special effort was made in handling animals to minimize stress or anxiety.

### Maternal separation protocol

MS protocol started at PND2 and finished at PND21. In long MS, offspring was separated from the dams for 3 h per day during the light period. The dams were removed from the home cage and placed in a new cage. Brief MS was identical but, instead of 3 h, dams and litters were separated for only15 min 3 times/day. After the MS period, animals were returned to the home cage. Animals from the unhandled group were left undisturbed except for litter weighing.

### Maternal behavior observations

Maternal behavior observations were conducted every day for 30 min after dams-pups reunification. To force retrieval, pups were removed from the nest and individually scattered across the home cage before the video recording. The following maternal behaviors were recorded: arched-back nursing, licking/grooming, pup retrieval (from P2 to P13 only) and latency to the first maternal behavior. The following non-maternal behaviors were recorded: eating, drinking and self-grooming. Finally, we also analyzed nest building, a behavior not exclusively maternal but clearly associated to caregiving in our study. Two independent observers separately scored maternal behavior.

### Adult behavioral analysis

Behavioral procedures were carried out as described previously (Boyer et al., 2019; Wu et al., 2021). All behaviors were analyzed in both male and females.

### Behavioral time line

All mice were tested following this specific timeline: day 1–3: affiliative touch (basal, control separation, stress); day 5: novel object recognition; day 7: olfactory testing; day 9: 3-chambers social task; day 11: intruder test; day 13: interactions in a neutral arena; day 15: openfield.

### Affiliative touch

For the allogrooming experiments, we used a protocol described elsewhere (Wu et al., 2021). Briefly, we first analyzed baseline allogrooming by recording interactions previous to any separation. The next day, the CD1 partner was removed from the home cage and placed into a separate clean cage. After 30 min, it was returned to the home cage to reunite with the subject (control separation). 24 h after, a second separation (stress) in which the CD1 partner was physically restrained for 30 min before being returned to the home cage was carried out. Videos from the experiments were manually annotated for different behaviors in a frame-by-frame manner. Allogrooming was defined as previously (Wu et al., 2021) (visible licking and/or mouth contact during which the C57Bl6J target mouse shows head bobbing indicative of licking motions and frequently holds the CD1 partner with forelimbs).

### Novel object recognition

For the novel object recognition task, we used the same arena as for the open field and small plastic objects. In the initial phase, the animals were confronted to two identical objects (green cylinders, 3.5 cm high, 4.5 cm in diameter). These objects were placed 10 cm from the wall and 5.5 cm apart from each other. The mice explored the arena for 10 min and were brought back to their cages for 24 h. In the second phase, one of the objects was replaced by a novel object (yellow triangular prism, 3.5 cm high, 4.5 cm on each side). The time spent exploring each object (nose point within 2 cm from the object) was quantified and used to calculate a Recognition Index (RI) as follows: RI = (time exploring object 1)/(time exploring object 1 + time exploring object 2).

### Olfactory testing

Mice were exposed to sequential presentations of different odors. The odor sequence used was water (reference), lemon and strawberry (non-social cues) and urine from males and females (social odors) embedded in a 2 cm × 2 cm piece of cotton. Each odor (or water) was presented in three consecutive trials for a duration of 2 min. The inter-trial interval was 1 min. We analyzed the time spent in sniffing the different odors (nose in close contact, <2 cm, to the olfactory stimulus).

### Three chamber social task

The three-chambers apparatus is a rectangular arena (60 cm × 37.5 cm × 21 cm) made of transparent plexiglass plastic, divided into three compartments of the same size (18.5 cm × 37 cm × 21 cm). Two openings connect the center chamber with the two side chambers. A cylindrical container (10 cm in diameter, 20 cm high) is present in each side-chamber. During the first trial (exploration), the target mouse was placed in the middle chamber facing a wall and allowed to explore. In the next trial (social), a stranger mouse was placed in one of the containers while the other was empty. In the last trial (novelty), the same mouse was placed on one side but a novel stranger was placed in the other container. Trials lasted 10 min and animals were left for 10 min in their home cages between trials. Strangers were juvenile CD1 male mice (3–5 weeks) that had been trained to be restrained in the container. Position of mice for each phase was randomized. For the analysis, chamber containing the mouse (during the social phase) and the novel mouse (novelty phase) were always considered the right chamber. A side-preference index (SPI) was calculated as follows: SPI = (time exploring right container)/(time exploring right + time exploring left container). To quantify the behavior, only the first 5 min of each trial were used.

### Intruder test

To avoid any aggressive behaviors, a juvenile CD1 male (3–5 weeks) was introduced into the home cage of the test mouse and recorded for 10 min. Different social (approach, avoidance, aggression or submission) and non-social behaviors initiated by the C57Bl6J mice were measured.

### Interactions in neutral arena

In this task, the target C57Bl6J mouse and a juvenile CD1 male (3–5 weeks) were simultaneously introduced into the open field arena as before. Different social and non-social behaviors (as in the intruder test) initiated by the target mice were measured.

### Open field

The open field device consisted of a non-reflective opaque plexiglass box (40 cm × 40 cm × 40 cm). To begin the test in the least stressful conditions, mice were placed facing a wall. Animals were recorded for 10 min and the following behaviors analyzed: time spent close to the walls (2.5 cm) or the arena center, time of immobility and total distance traveled.

### Behavioral data acquisition and quantification

All experiments were video recorded (Ethovision XT, Noldus, Netherlands) and analyzed offline. For tests involving two freely moving animals, an investigator blind to the conditions manually scored the behaviors. For the other tests, mice tracking and behavioral analysis were carried out using dedicated software (Ethovision XT).

### Statistical analysis

Data are presented as group mean ± standard error of the mean (SEM). In bar graphs, individuals are represented as dots symbols. GraphPad Prism 9 (GraphPad Software Inc., CA, USA) was used to perform all statistical analysis. Due to the limited sample size, normality of the data was evaluated using kurtocity and skewness of the distributions (see Supplementary material). To investigate the alterations in maternal care by the different MS protocols, we performed 1-way ANOVA followed by Dunnett’s *post hoc* tests. For temporal analysis, we used 2-way ANOVA for repeated measures and Tukey’s test. For adult behavioral analysis, 1-way or 2-way ANOVA were used. All statistics and tests used are provided as Supplementary material. Value of *p* < 0.05 was considered as statistically significant.

## Results

### Differential effects of brief versus long MS effects on dam behavior

The first aim of our report is to provide a comprehensive characterization of the MS effects on maternal behavior. We compared two protocols of MS; in long MS (LMS), the dam was placed in a clean cage for 3 h/day whereas brief MS (SMS) consisted on three episodes/day lasting 15 min each. As a control, unhanded litters were used. We then quantify maternal and non-maternal behavior upon reunification.

**Figure 1:**
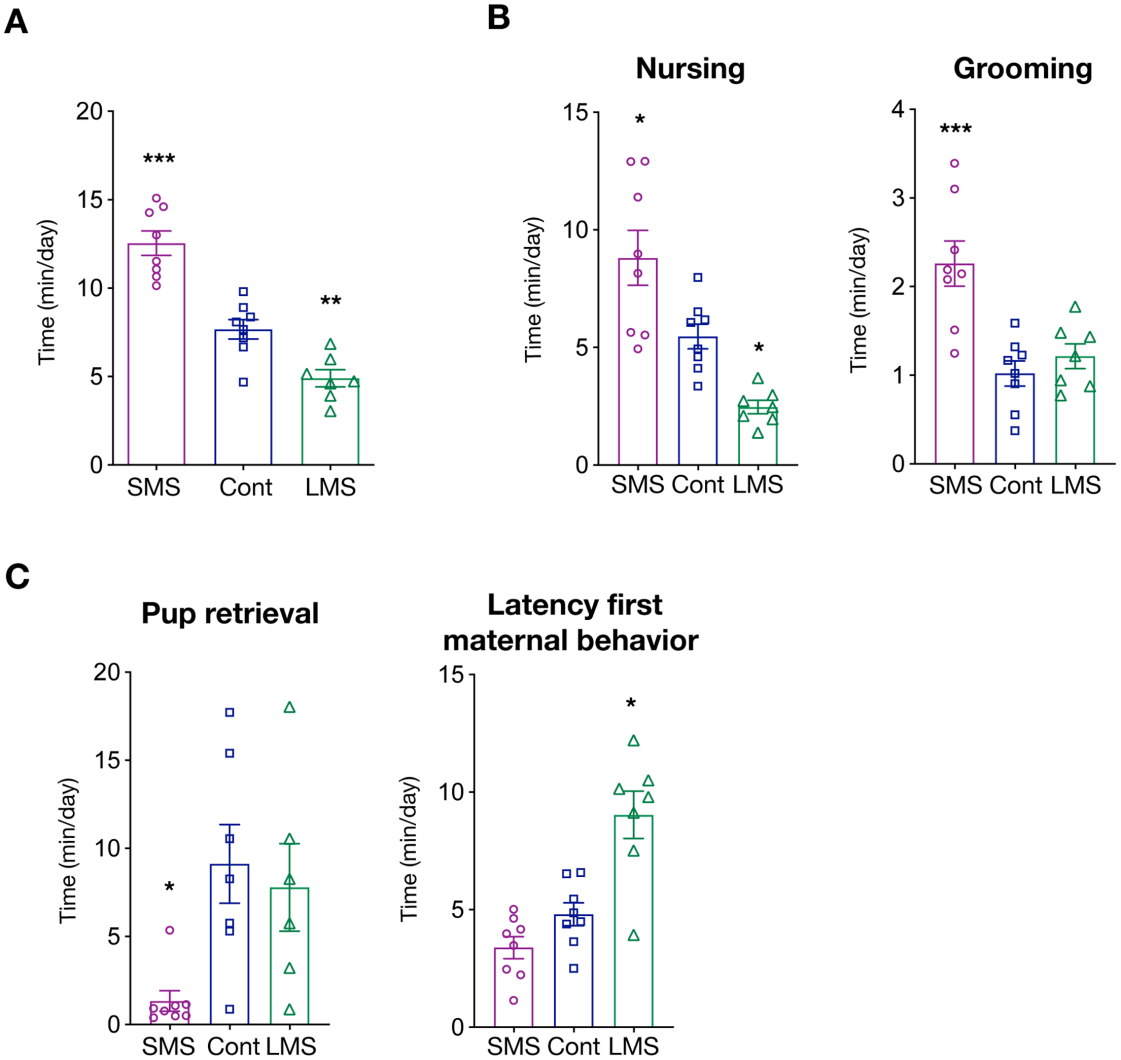
Effects of short and long MS protocols on maternal behavior. **(A)** Total time spent in active maternal caregiving. SMS and LMS resulted in a significant alteration of total time of active maternal behaviors compared to unhandled controls (*n* = 8 for SMS and controls, *n* = 7 for LMS). **(B)** Time spent in active nursing and litter grooming. Both SMS and LMS affected nursing time (left panel). SMS also increased grooming (*n* = 8 for SMS and controls, *n* = 7 for LMS). **(C)** Time spent in other maternal behaviors. Effects of SMS and LMS on necessary to retrieve the litter and the latency for the first active maternal behavior (*n* = 8 for SMS and controls, *n* = 7 for LMS). * *p* < 0.05, ** *p* < 0.001, *** *p* < 0.0001.

We first measured total time spent in maternally-related behaviors during the whole post-natal period (PND2-20). As expected, MS had a strong influence in dam caregiving levels (*p* < 0.0001) (Figure 1A). More precisely, brief MS significantly increased the time of active dam maternal behaviors whereas long MS had the opposite effect (*p* < 0.00001 SMS vs. control; *p* < 0.01 LMS vs. control).

We then investigated how MS affected specific maternal behaviors. We focused first on arched-back nursing (from here, nursing), an essential maternal behavior for optimal pup feeding, and maternal grooming, as it provides the first social interactions. Our results showed that there is a strong influence of MS on the time spent on nursing (*p* < 0.0001) (Figure 1B). SMS resulted in an enhanced maternal nursing while long MS reduced the average time of nursing (*p* < 0.05 SMS vs. control; *p* < 0.05 LMS vs. control). We also quantified nursing initiated, not by the dam, but upon pup demand (passive maternal care). Interestingly, as depicted in Supplementary Figure 1A, no difference could be detected among conditions suggesting that MS might influence primarily maternal drive. Litter weight was identical across conditions suggesting that, although providing different social environment, feeding is either not severely impaired or compensated by pup active breast seeking (Supplementary Figure 1B).

We also quantified the average time per day that the dam spent in pup grooming (a combination of social touch and licking) (Figure 1B). Our findings uncovered a highly significant effect of MS (*p* < 0.0001). Remarkably, post-hoc multiple comparison analysis revealed that this is mainly driven by a dramatic increase in the brief MS group (*p* < 0.001). Long MS had no effect on dam grooming (*p* = 0.7030).

To explore in further detail the consequences of MS, we analyzed other features of maternal caregiving including the time required for pup retrieval or the latency to the first maternal behavior (Figure 1C). We observed that MS profoundly altered those behaviors but, again, that the consequences were not identical between both types MS. LMS significantly increased the latency for the appearance of the first maternal behavior (*p* = 0.2378 SMS vs. control; *p* < 0.001 LMS vs. control) whereas the time required for pup retrieval was only reduced by SMS (*p* < 0.05 SMS vs. control; *p* = 0.8361 LMS vs. control).

Finally, we investigated other dam behaviors independent of caregiving such as drinking, eating or self-grooming (Supplementary Figure 1C). We observe a difference in the time spent in drinking behavior of dams submitted to LMS (*p* = 0.0029 LMS vs. control). MS did not influence eating (*p* = 0.0926) or self-grooming (*p* = 0.2867). Finally, we also assessed nest building, a behavior that, although present independently of the offspring, is largely increased in lactating dams. We found no influence of MS on the time spent in nest building (Supplementary Figure 1D). Overall, our data indicates that separation from the litter largely impinge on maternal behaviors.

### Time course analysis of MS effects on dam behavior

**Figure 2:**
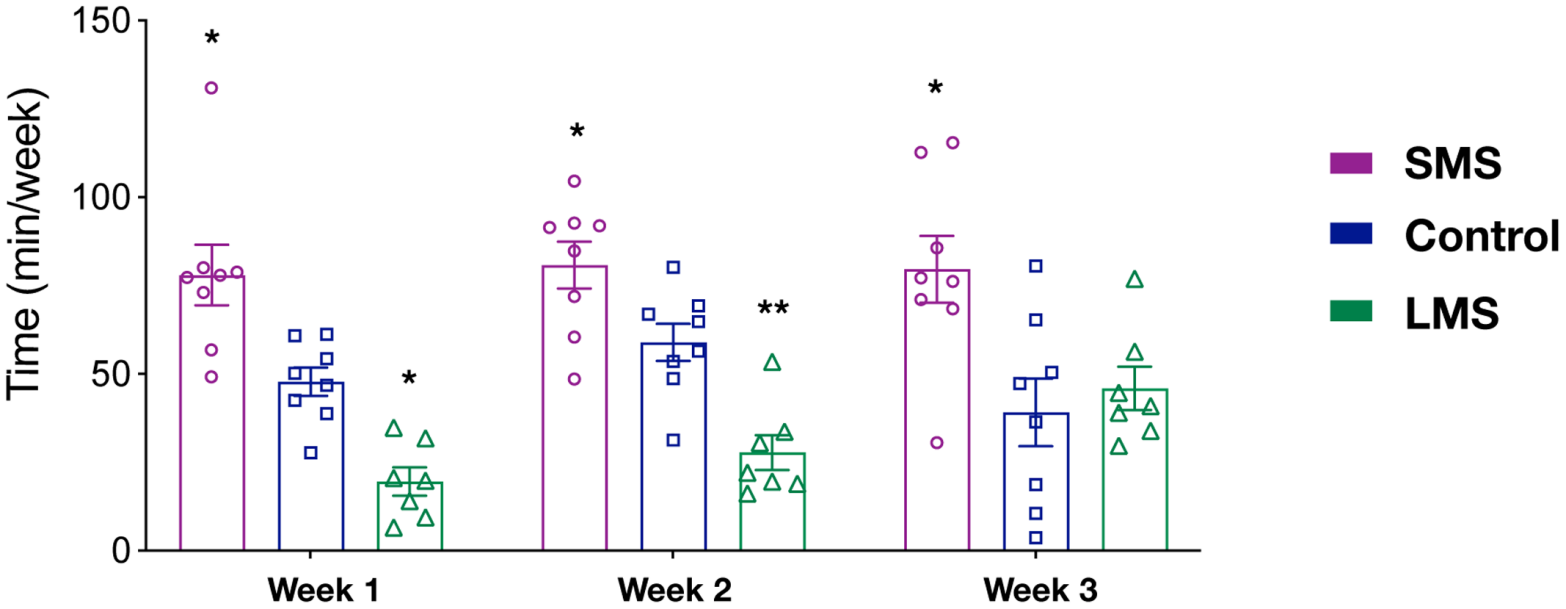
Temporal analysis of the effects of short and long MS protocols on total active maternal behavior. The time spent in active maternal caregiving was calculated for the first three post-natal weeks. SMS led to a significant increase across the entire post-natal period. LMS reduced this time and this effect was only present in the early post-natal period week (*n* = 8 for SMS and controls, *n* = 7 for LMS). * *p* < 0.05, ** *p* < 0.001.

Previous reports have suggested that the effects of MS on the offspring critically rely on the timing of MS. However, it is not clear whether MS influenced maternal behavior in a time dependent manner. As an indirect approach, we sought to analyze our data in 3 week-blocks (PND2-8; PND9-14; PND15-20) (Figure 2). We observed that there was a significant effect of MS (*p* < 0.0001) but no effect of time (*p* = 0.406) in the total time spent in maternal behaviors. Although there was not a significant interaction of MS and time (*p* = 0.091), we observed that SMS dams spent significantly more time in active maternal care during the whole post-natal period (*p* < 0.01 week 1; *p* < 0.05 week 2; *p* < 0.05 week 3). In contrast, LMS differences were observed during the first 2 weeks (*p* < 0.05 week 1; *p* < 0.01 week 2; *p* = 0.933 week 3). These findings suggest that the effects of brief and long MS are also different in terms of temporal dynamics.

To provide further support to this hypothesis, we carried out a similar temporal analysis on other behavioral parameters (Supplementary Figure 2). Regarding grooming, we found a significant effect of time (*p* < 0.05). Thus, grooming increased significantly in all groups from the first to the second week (*p* < 0.01). We also observed a significant effect of MS (*p* = 0.0003). As shown in the previous analysis (Figure 1B), this resulted from a sustained upregulation of grooming in the SMS group (Supplementary Figure 2A). We found no interaction of both factors (*p* = 0.635).

For the nursing behavior, we could only detect an effect of MS (*p* < 0.0001) but not of time (2-way ANOVA repeated measures, *F*(2, 20) = 1.814; *p* = 0.176). There was no time x MS interaction (*p* = 0.352; Supplementary Figure 2B). Together, these data indicate that SMS and LMS show a completely different temporal pattern and that the effects of LMS on maternal behavior are almost absent at the end of the post-natal week 2.

### Effects of maternal environment on the offspring behavior at adulthood

We next investigated whether these different rearing environments resulted in long lasting behavioral differences. For that, litters being exposed to brief or long MS protocol as well as unhandled controls were tested in several paradigms exploring social and non-social functions (see Section “Methods”).

**Figure 3:**
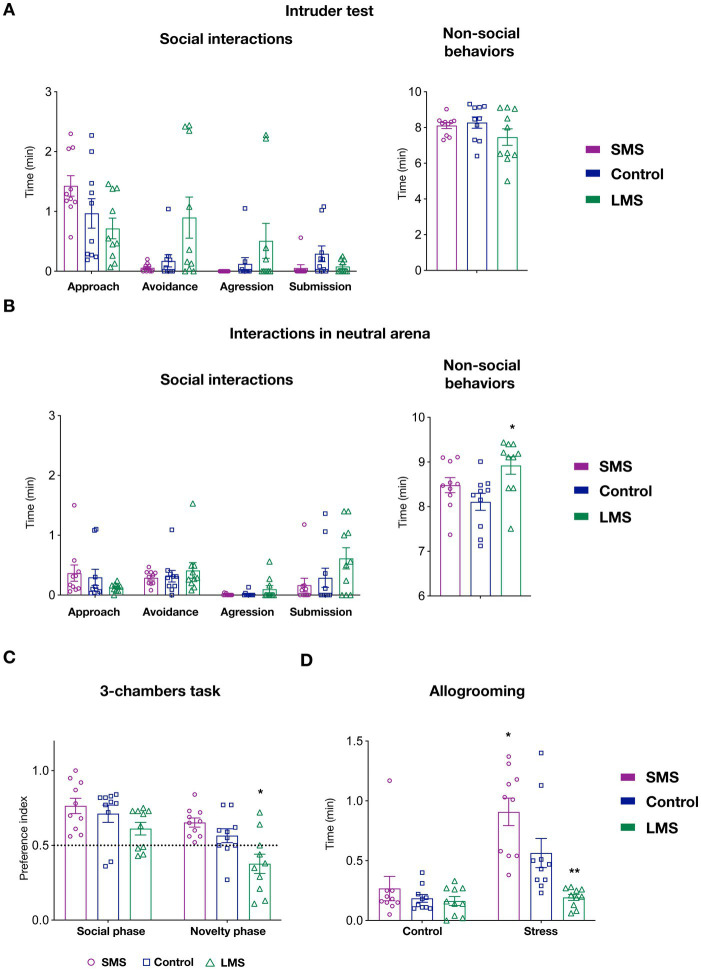
Effects of short and long MS protocols on offspring behavior. **(A)** Intruder test. Left panel show the effects of SMS and LMS in social behaviors (approach, avoidance, aggression and submission). Right panel display time spent on non-social behaviors during the task (*n* = 10 mice/group). **(B)** Interactions with a juvenile mouse in a neutral arena. Left panel show the effects of SMS and LMS in social behaviors (approach, avoidance, aggression and submission). Right panel display time spent on non-social behaviors during the task (*n* = 10 mice/group). **(C)** 3-chambers social task. In this test, mice explore an arena containing a mouse and empty restrainer (social phase) or a familiar and a novel mouse (novelty phase). When the preference index (see Section “Methods”) was quantified, we observed no effect of SMS. In contrast, LMS decreased the preference for the novel mouse during the novelty phase. (*n* = 10 mice/group). **(D)** Affiliative touch. In this test, allogrooming of the subject mouse toward the cage mate was quantified after a 30 min separation. In the control phase, the partner is replaced in a separate cage whereas in the stress phase (24 h later) the partner is submitted to physical retrain. As previously shown, control mice increase allogrooming in response to partner’s distress. This effect was enhanced in the SMS group and abolished by LMS (*n* = 10 mice/group). * *p* < 0.05, ** *p* < 0.001.

Since maternal care provide the first social environment, we reasoned that MS might likely have an impact on the development of socially-related behaviors. We therefore tested, animals raised under different maternal conditions in multiple social paradigms. We first performed the intruder test (Figure 3A). In this task, we detected no effect of maternal separation on social (*p* = 0.3145) or non-social behaviors (*p* = 0.2201). Despite of being infrequent, some animals exhibited an aggressive behavior toward the intruder in both the control and, especially, in the LMS group. Remarkably, none of the animals in submitted to SMS showed aggression.

We next measured social interactions with a juvenile mouse in a neutral arena (Figure 3B). We could not observe either a significant effect of MS in any of the social behaviors scored (approach, avoidance, submission or aggression) (*p* = 0.3464). Similar to the previous task, we observed that SMS mice never exhibited an aggressive behavior. Finally, there was a statistical difference in the time spent on non-social behaviors in this test (*p* < 0.05) (Figure 3A). Specifically, mice in the LMS group showed an increase in the duration of these behaviors (*p* < 0.05) whereas those in the brief MS did not (*p* = 0.342).

In the 3-chambers social task, 2-way ANOVA revealed a very significant interaction of the maternal environment and the side-preference ratio during the social and novelty phases (*p* < 0.001). Our data indicated that mice in the SMS group exhibited the expected preference for the social partner in the social phase and for the unfamiliar conspecific during the novelty phase (Figure 3C). No difference can be detected in these mice compared to the unhandled controls (*p* = 0.6844 social phase; *p* = 0.2763 novelty phase). Long MS did not affect the choice during the social phase but significantly decreased the preference for the unfamiliar mouse in the novelty phase (*p* = 0.6844 social phase; *p* < 0.05 novelty phase).

We also sought to investigate the consequences of different MS on allogrooming, a behavior linked to the perception of conspecific stress (Wu et al., 2021). For that, we measured the time that C57Bl6J mice on the different groups spent in grooming their CD1 cage mate after 30 min of separation. We observed that MS strongly influenced this test (*p* < 0.01). Our data on unhandled control animals (Figure 3D) confirmed previous observations and showed a significant increase of affiliative touch when the partner mouse had been previously stressed (*p* < 0.01, control vs. stressed phase). When we performed a multiple comparison analysis, we found no change in affiliative touch among the three groups during the control phase but a significant difference in the response to the stressed cage mate (*p* < 0.01, control vs. long MS; *p* < 0.01, control vs. brief MS). These findings indicate that the most robust effects of MS impinge on the neural circuits processing recognition of emotional status.

To explore whether MS also modulated non-social behaviors, we evaluated our mice on other behavioral tasks. Since social behavior in mice relies on olfactory cues, we first investigated whether MS altered odor perception. We analyzed the sensitivity to different olfactory cues including urine (from the same or opposite sex) as well as the desensitization after 3 exposures to the same odorant. When analyzed together, we find no difference in the responses to different olfactory stimuli among our three groups of animals (*p* = 0.0810; Supplementary Figure 3). To further ensure that there is no olfactory alteration, we analyzed responses to individual odorants and, again, we did not observe any significant change (water *p* = 0.84; vanilla *p* = 0.148; lemon *p* = 0.215; same sex urine *p* = 0.379; other sex urine *p* = 0.306) confirming that MS did not alter olfaction and that the observed differences in social behavior did not result from olfactory dysfunction.

We also examined the performances in the open field. In this test, we assessed locomotion (by measuring the total distance traveled), the exploration patterns (by quantifying the time spent in immobility) and the presence of anxiety-like behaviors (by analyzing the proportion of time in the center of the arena versus the wall zone). MS has no effect on any of those behaviors (Supplementary Figure 3B) except in the time spent in the center of the openfield (*p* < 0.05) where animals of the SMS group exhibited an increased time (*p* < 0.05 SMS vs. control) indicative of decreased anxiety.

Lastly, we evaluated cognitive performances and memory in the novel object recognition task (Supplementary Figure 3C). As expected, unhandled controls explored similarly the two objects during the first trial and spent significantly more time around the novel object in the second one. We observed that MS did not modify the performances in the first phase (*p* = 0.87) but resulted in significant changes during the second phase (*p* < 0.05). Multiple comparison revealed that the performances of the brief MS group were comparable to those of the control mice (*p* = 0.987). The group of long MS showed no preference for the novel object in the second trial and that was significantly different compared to control group (*p* < 0.05).

## Discussion

The aim of the present study was to examine, in detail, the behavioral consequences of two models of MS that have been used extensively in rodents. Our results indicated that variations in postnatal separation not only influenced the extent and quality of maternal care provided by the dam but also the social behaviors of the offspring at adulthood. Overall, we found that LMS decreased caregiving and impacted negatively the behavioral performances of the progeny whereas SMS had a positive effect. Finally, our findings pointed out maternal care levels impinge primarily on the responses of the offspring to conspecific stress (allogrooming, Figure 3D) and the absence of aggression toward unfamiliar conspecific (Figures 3A,B).

It is important to highlight that, in our report, we intentionally introduce two major changes compared to classical MS protocols (see Section “Methods”). On one hand, our brief MS consisted of three rather than a single episode of 15 min separation per day [see for example (Singh-Taylor et al., 2018)]. Our aim was to reproduce a more naturalistic setting as it has been long known that lactating dams leave the nest for food seeking several times a day (Grota and Ader, 1974; Hughes et al., 1978). On the other hand, we housed the offspring with old non-reproductive CD1 females after weaning. Although most studies use littermates as cage partners, we strongly believe that CD1 females provide a more controlled social environment. First, if left with littermates, all mice are housed with animal from the same sex. Since males and females might not show the same interactions, a CD1 female provides an homogeneous social context and, more importantly, equivalent for all mice. Second, CD1 females retired from breeding are known to display much higher levels of social tolerance limiting therefore aggressive behaviors exhibited by C57Bl6 mice (especially males). A potential disadvantage of this co-housing strategy is that females and males are exposed to same-sex and other-sex interactions, respectively. Although this can be a potential confounding factor, we should highlight that, because of their age, retired breeders represent a poor mating stimulus and might be therefore a sexually-neutral social partner. We are convinced that these methodological improvements, although not perfect, may be useful for future studies.

Although proposed to recapitulate early-life traumatisms, most of the studies have only reported minor behavioral changes in the offspring after MS [reviewed for example in Orso et al. (2019)]. According to the existing literature, an increase in anxiety-related behaviors is the most consistent alteration observed following MS, especially in the extended versions (similar to our LMS) (Orso et al., 2020; Tsotsokou et al., 2021; Wang et al., 2022). However, other studies reported no such effect (Tan et al., 2017), a differential outcome depending on the behavioral test used (van Heerden et al., 2010) or even decreased anxiety (Venerosi et al., 2003). The lack of consistent phenotypes upon MS protocols may arise from multiple factors. Among them, the differences in separation time might be crucial. To address this issue, we sought to simultaneously carry out two different paradigms (long and short MS) so that we can provide experimental evidence of their differential effects (or not). We found that both MS protocols profoundly altered maternal behavior and therefore offspring caregiving. More importantly, SMS and LMS resulted in overall opposite consequences confirming previous reports (Singh-Taylor et al., 2018). In agreement with prior findings, we detected behavioral impairments only in mice exposed to low levels of maternal care, which might mimic infant neglect in humans. Although mostly associated to social tasks (interactions in neutral arena, allogrooming and 3-chambers task), LMS has also some impact on cognitive tasks such as the novel object recognition. In contrast, a positive maternal environment provided by LMS resulted in no obvious deficit but in enhanced affiliative responses to the stressed partner. Together, our data pointed out the long-known deleterious effect of a negative early social context on the brain under construction (Tottenham, 2020) and also extend the notion that an enriched environment can promote emotional functioning and, eventually, resilience. A future study that will investigate the effects of LMS and SMS in vulnerability to adult stress (i.e., chronic social defat) is planned.

Previous work has suggested that MS effects depend on the precise time window in which they are applied. Thus, Peña et al. showed that long MS during the post-natal period (PND1-20) enhances susceptibility to adult stress (Peña et al., 2017). The authors observed similar effects of shorter MS during the late post-natal period (PND10-20). Interestingly, early MS (PND2-12) did not increase the susceptibility to develop depression-related behavioral abnormalities in response to adult stress arguing for the existence of critical periods for MS. Although we did not directly tested the effects of MS at different time windows (e.g., P2-P10 vs. P11-20), our observations suggest that LMS impinge on maternal care levels in the early post-natal period and that this effect tend to fade away later (Figure 2; Supplementary Figure 2). This discrepancy might arise from the experimental design (Peña et al did combine MS and limited bedding) or simply reflect that maternal care levels do not participate in the susceptibility to adult stress. Again, ongoing work on the effects of MS to adult stress would shed some light into this issue.

One of the major findings in this work is that maternal care levels bidirectionally modulated the offspring ability to react to conspecific distress. To the best of our knowledge, this is the first time that MS has been shown to impact affiliative behaviors. Thus, SMS increased allogrooming whilst animals in the LMS group failed to show affiliative touch in response to the stressed cage mate. This observation may be very relevant for translational research as numerous evidences in humans suggest that early-life traumatic events are not only associated to a higher risk of psychopathology but selectively impair recognition of emotion in others (Bérubé et al., 2023). Although the neural circuits implicated in such behaviors have begun to be studied in rodents (Wu et al., 2021), our findings suggest that early MS could be instrumental to gain insights into the underlying neural mechanisms. Thus, one could study whether absence of allogrooming after LMS results from impairments in perceiving a negative emotional state in conspecifics, from a decreased emotional responsiveness to partner’s distress or from both.

Certain limitations should be considered when interpreting the current work. One caveat is that we purchased all pregnant females. It has been known that transport could influence embryonic development and increase the variability of MS effect. This can act as an important confounding variable and limit our ability to detect subtle behavioral changes. In addition, it makes more difficult to compare our study to many other previous reports in which animals were bred in-house. However, it provides an obvious advantage for replicating our finding in future studies. Second, to the best of our knowledge, we are the first to describe an effect of MS in allogrooming. Further studies are needed to assess if these results are reproducible. Third, compared to co-housing animals with littermates, our choice of an old non-reproductive female as social partner could result in a severe reduction of playing during the adolescence period. Given the important role of playing on mammalian brain development (Shaheen, 2014; Pellis et al., 2023), future studies might consider a mixed co-housing approach, starting with littermates and moving to old females later on. Finally, to reduce the number of mice [3Rs principle (Flecknell, 2002)], our study design involved a battery of test to be performed in all animals for a duration of 2 weeks. Although animals were exposed from less to most stressful tests to minimize the impact on behavioral results, we can not rule out that this extended testing has an influence in the performance on individual tasks.

In conclusion, although extensively used, the effects of MS in mice as well as their potential use in translational research are not fully elucidated. We believe that the detailed behavioral analysis of both dam and offspring presented in this study may be of great interest for those researchers aiming at mimicking the characteristics of human early life adverse conditions in reliable rodent preclinical models.

## Data availability statement

The original contributions presented in the study are included in the article/Supplementary material, further inquiries can be directed to the corresponding author.

## Ethics statement

The animal study was approved by Cefos local ethics committee CE14 (EU31477) under the following protocol (#31477-2020021219331839 v7). The study was conducted in accordance with the local legislation and institutional requirements.

## Author contributions

CR: Conceptualization, Data curation, Formal analysis, Investigation, Writing – original draft. DR-M: Data curation, Formal analysis, Investigation, Writing – original draft. CL: Formal analysis, Investigation, Writing – review & editing. EG: Conceptualization, Funding acquisition, Methodology, Project administration, Supervision, Writing – original draft.
