# Supplementary material for: Brief and long maternal separation in C57Bl6J mice: behavioral consequences for the dam and the offspring
Source: Front Behav Neurosci. 2023 Oct 23;17:1269866. doi: 10.3389/fnbeh.2023.1269866 (PMC10626007; doi:10.3389/fnbeh.2023.1269866)
Supplement: Supplementary file 4 [file Image_1.pdf]

Rombaut et al Suppl Figure 1

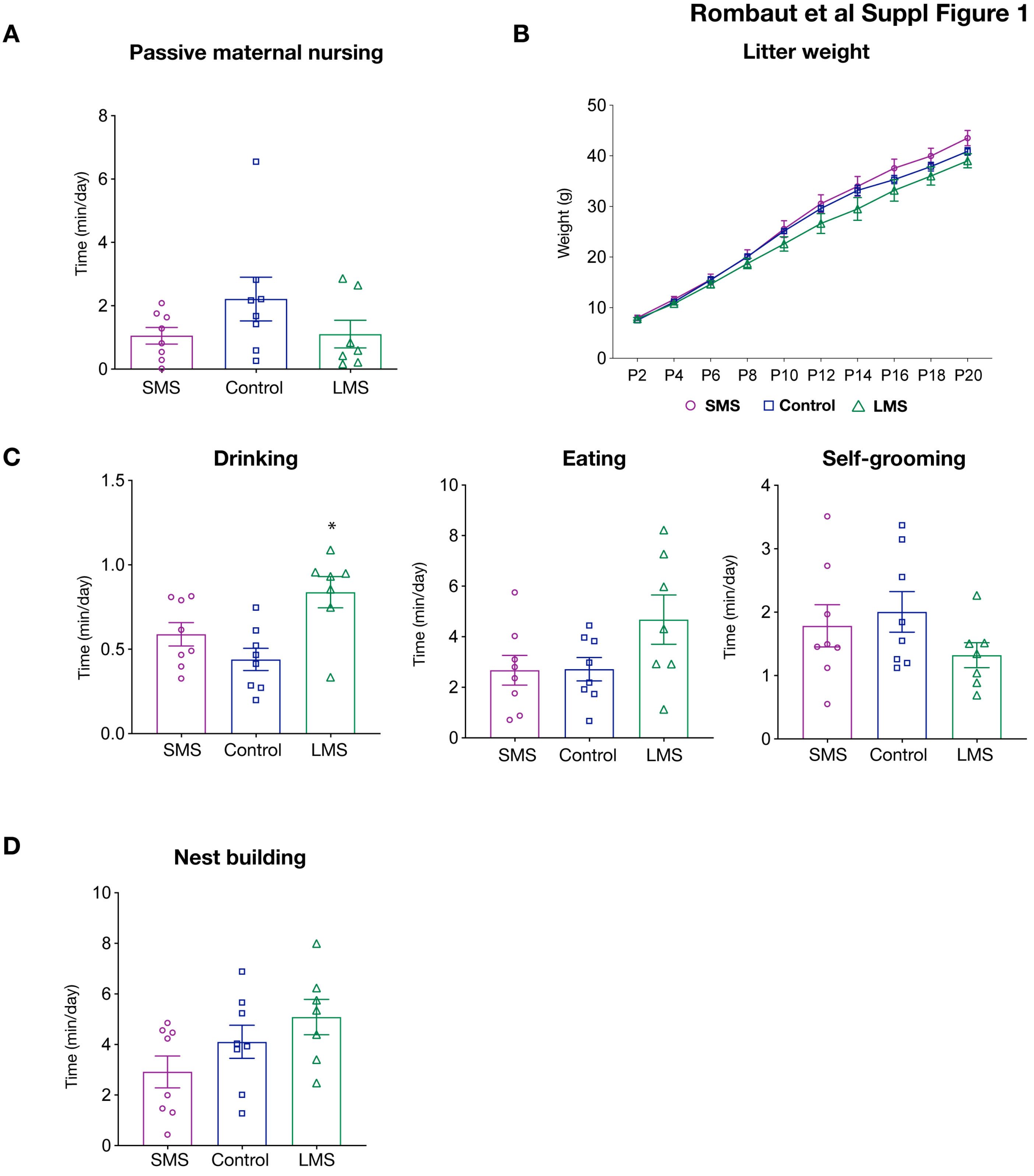

Rombaut et al Suppl Figure 2

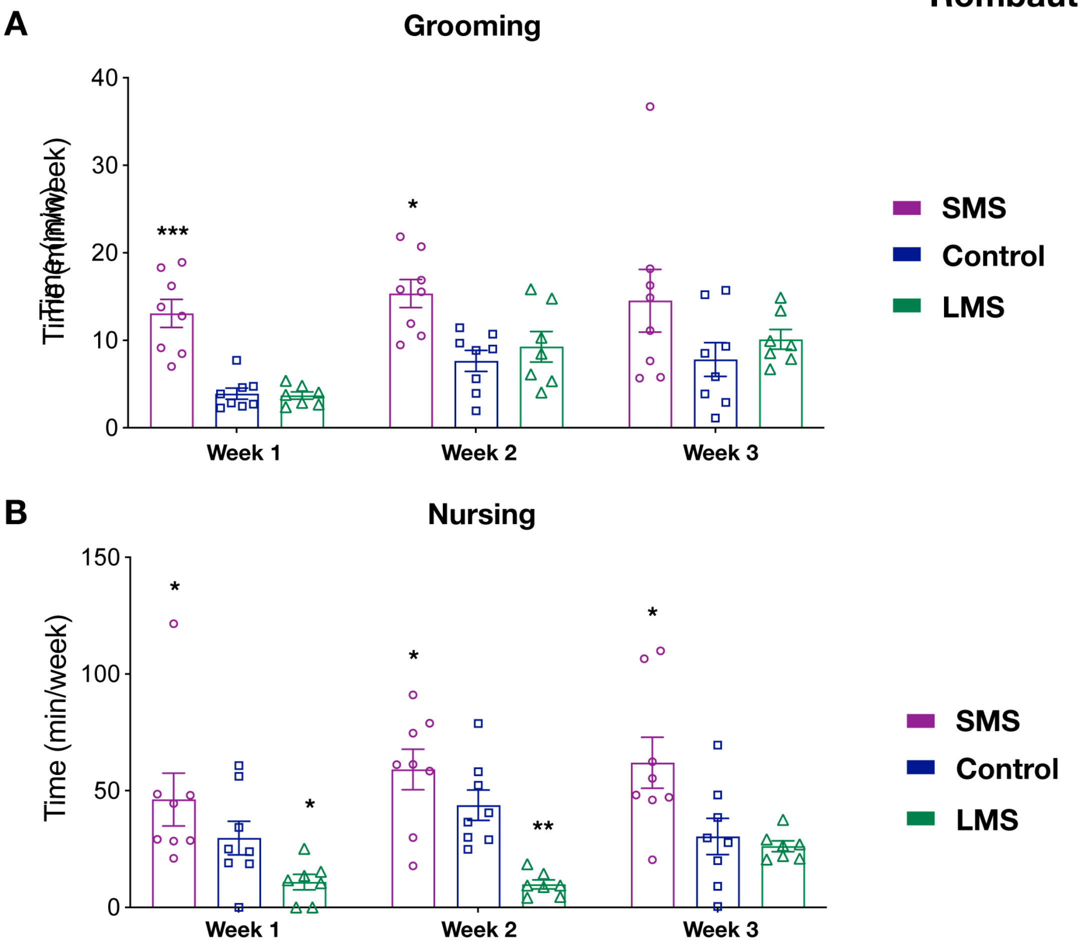

Rombaut et al Suppl Figure 3

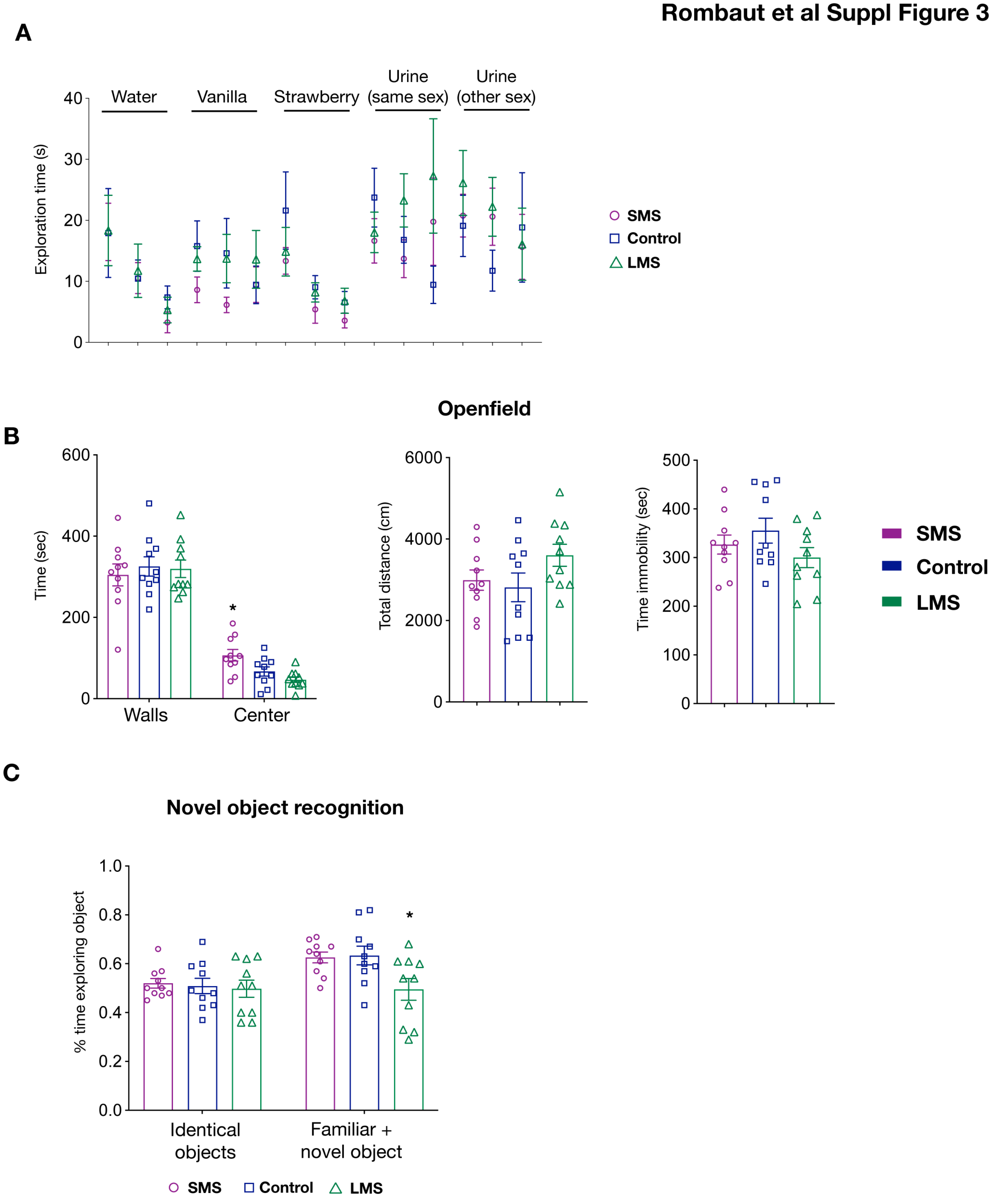

SUPPLEMENTARY FIGURE LEGENDS

Figure 1. Effects of short and long MS protocols on non-maternal behavior.

A. *Total time spent in nursing behavior initiated upon pup demand (passive maternal care)*
B. *Litter weight across post-natal development. SMS and LMS did not significantly alter growth curves.*
C. *Time spent in drinking, eating and self-grooming (n=8 for SMS and controls, n=7 for LMS).*
D. *Time spent in nest building, a behavior that, although not intrinsically maternal, is highly associated to pup caregiving.*

   ** $p<0.05$*

Figure 2. Temporal analysis of the effects of short and long MS protocols on other maternal behaviors.

A. *Total time spent in grooming.*
B. *Total time spent in nursing.*
C. * $p<0.05$; ** $p<0.001$; *** $p<0.0001$

Figure 3. Effects of short and long MS protocols on offspring non-social tasks.

A. *Olfactory function was not affected by MS. (n=10 mice/group)*
B. *Analysis of behavior in the open field. Left panel: effects of SMS and LMS in the time spent near the walls or in the center of the arena. Middle panel: total distance traveled in mice raised in different environments. Right panel: time of immobility (n=10 mice/group)*
C. *Novel object recognition task. In this test, mice explore two identical objects or a familiar and a novel object. A preference index (see methods) was quantified. We observed no effect of SMS. In contrast, LMS decreased the investigation preference for the novel object. (n=10 mice/group).*

   ** $p<0.05$*
